# Evolvability and constraint in the evolution of three‐dimensional flower morphology

**DOI:** 10.1002/ajb2.16092

**Published:** 2022-11-13

**Authors:** Øystein H. Opedal, Laura S. Hildesheim, W. Scott Armbruster

**Affiliations:** ^1^ Department of Biology Lund University Sölvegatan 37 22362 Lund Sweden; ^2^ School of Biological Sciences University of Portsmouth Portsmouth PO1 2DY UK; ^3^ Institute of Arctic Biology University of Alaska Fairbanks Fairbanks AK 99775 USA

**Keywords:** conditional evolvability, *Dalechampia*, Euphorbiaceae, functional constraints, genetic constraints, plant–pollinator interactions

## Abstract

**Premise:**

Flower phenotypes evolve to attract pollinators and to ensure efficient pollen transfer to and from the bodies of pollinators or, in self‐compatible bisexual flowers, between anthers and stigmas. If functionally interacting traits are genetically correlated, response to selection may be subject to genetic constraints. Genetic constraints can be assessed by quantifying standing genetic variation in (multivariate) phenotypic traits and by asking how much the available variation is reduced under specific assumptions about phenotypic selection on functionally interacting and genetically correlated traits.

**Methods:**

We evaluated multivariate evolvability and potential genetic constraints underlying the evolution of the three‐dimensional structure of *Dalechampia* blossoms. First, we used data from a greenhouse crossing design to estimate the **G** matrix for traits representing the relative positions of male and female sexual organs (anthers and stigmas) and used the **G** matrix to ask how genetic variation is distributed in multivariate space. To assess the evolutionary importance of genetic constraints, we related standing genetic variation across phenotypic space to evolutionary divergence of population and species in the same phenotypic directions.

**Results:**

Evolvabilities varied substantially across phenotype space, suggesting that certain traits or trait combinations may be subject to strong genetic constraint. Traits involved functionally in flower‐pollinator fit and autonomous selfing exhibited considerable independent evolutionary potential, but population and species divergence tended to occur in phenotypic directions associated with greater‐than‐average evolvability.

**Conclusions:**

These results are consistent with the hypothesis that genetic constraints can hamper joint trait evolution towards optimum flower‐pollinator fit and optimum self‐pollination rates.

Evolution of complex phenotypes is commonly governed by multiple conflicting selection pressures interacting with genetic constraints (e.g., Cheverud, 1984; Arnold, 1992; Walker, 2007; Futuyma, 2010). Flowers, for example, are complex three‐dimensional structures for which proper functioning (e.g., pollen transfer) depends on the precise coordination of multiple organs. Bisexual flowers and inflorescences face the challenge of ensuring fitness through both sexual functions, which requires organizing the sexual organs (anthers and stigmas) in ways that optimize pollen transfer, while at the same time avoiding interference between the male and female functions (Webb and Lloyd, 1986; Armbruster et al., 2009a, 2009b). Efficient pollinator‐mediated pollen transfer requires pollen deposition by anthers onto those parts of the pollinator's body that will be contacted by stigmas of subsequently visited conspecific flowers. In species where pollinators orient themselves in a systematic way during floral visits, the anthers and stigmas must be positioned at similar distances from the landmark that determines the position of the pollinator, such as a nectary or the walls of the corolla tube, at least in systems in which there is not much pollinator movement after landing (Armbruster et al., 2009b; Armbruster, 2014). Efficient pollen transfer can thus impose strong, and potentially conflicting, selection pressures on floral architecture.

Further conflicting selection pressures on flower architecture arise from the fact that, in self‐compatible species, compatible pollen arrives on stigmas through both within‐flower and between‐flower pollen transfer. These processes are only semi‐independent because efficient within‐flower pollen transfer may depend on pollinator visits (facilitated autogamy; Lloyd and Schoen, 1992) and because excessive self‐pollination may preclude subsequent pollinator‐mediated pollen deposition (e.g., Smith and Rausher, 2007). The efficiency of pollen transfer within flowers is strongly related to variation in herkogamy, the distance between male and female reproductive organs (Webb and Lloyd, 1986; Opedal, 2018). Increased herkogamy is generally thought to favor outcrossing because it reduces self‐pollen deposition and interference between pollen import and export (Webb and Lloyd, 1986). However, if pistils and stamens are oriented along a single dimension, increased herkogamy will also be associated with divergence between the distance of the anthers from the site of reward production (e.g., a nectary) and the corresponding distance for the stigmas (i.e., reward to stigma distance ≠ reward to anther distance, see Figure 1). Because these distances will usually determine the position of pollinator contact with the sexual organs, greater herkogamy can thus lead to reduced accuracy of pollen transfer due to greater deviation from the optimum phenotype for pollen transfer (Armbruster et al., 2009a).

**Figure 1:**
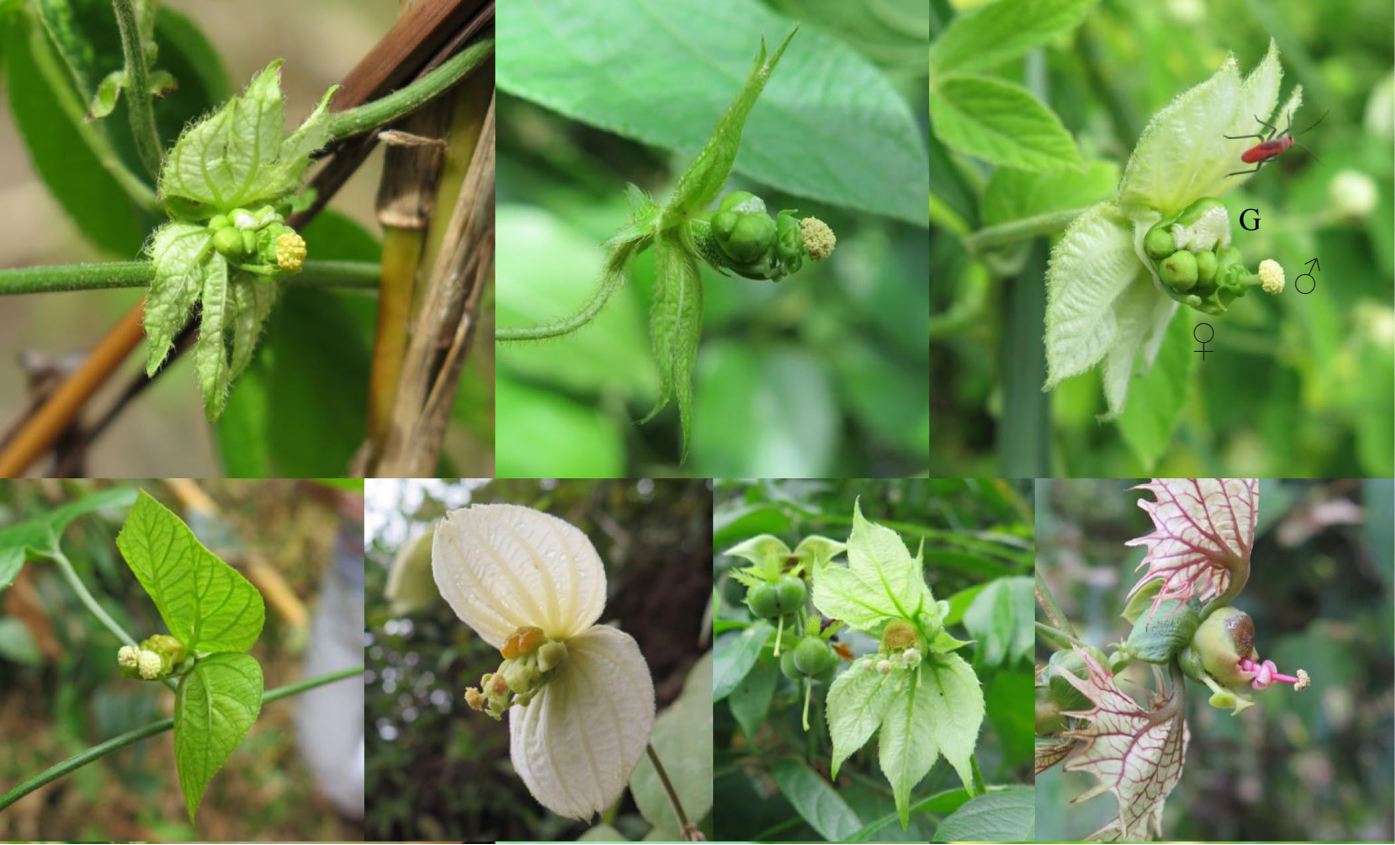
Blossom inflorescences of a selection of *Dalechampia* species, illustrating variation in the orientation of male and female flowers. All species shown have three female flowers (♀) situated below a cluster of ~10 male flowers (♂) and a resin gland (G) secreting colorless, yellow, or maroon resin. The top row shows three representatives of the *D. scandens* complex, with (left to right) zero, moderate, and pronounced herkogamy. The rightmost blossom belongs to the taxon used in our breeding design and population‐divergence analysis. The bottom row illustrates similar variation across the genus, in order of increasing mean herkogamy (left to right): *D. heteromorpha*, *D. tiliifolia*, *D. websteri*, *D. dioscoreifolia*. Note that species toward the left have male and female organs oriented along a single dimension, while sexual organs of species towards the right deviate at an angle.

If functionally interacting traits are genetically correlated, response to selection may often be subject to genetic constraints (Lande, 1979; Cheverud, 1984; Arnold, 1992; Blows and Hoffmann, 2005; Walker, 2007; Hansen and Houle, 2008; Walsh and Blows, 2009; Futuyma, 2010; Conner et al., 2011). Evolutionary quantitative genetics provides a straightforward theoretical framework for analyzing such constraint, emphasizing the key role of the additive genetic variance as a measure of evolvability (evolutionary potential). Available data on the evolvabilities (sensu Houle, 1992; i.e., mean‐scaled additive genetic variances) of individual traits suggest that lack of genetic variation in simple traits is unlikely to constitute a strong evolutionary constraint (Opedal, 2019; Hansen and Pélabon, 2021). However, less is known about the multivariate evolvability of complex phenotypes such as flowers, as summarized by genetic variance–covariance matrices (**G**). Studies of **G** matrices for complex phenotypes have revealed that genetic variance is often unevenly distributed across phenotype space, so that some phenotypic axes (trait combinations) are associated with above‐average evolvability, while others exhibit little or no evolvability (Gomulkiewicz and Houle, 2009; Hine et al., 2014; Blows et al., 2015; Blows and McGuigan, 2015). If selection operates on less evolvable trajectories in phenotypic space, genetic constraints can be severe.

If genetic constraints due to the genetic architecture of complex phenotypes are evolutionarily important, we expect patterns of population and species divergence to align with axes of high multivariate evolvability. Previous studies assessing such relationships are consistent with an important role of constraints in that they have revealed moderate to strong relationships between population divergence and evolvability quantified as standing variation within populations (Andersson, 1991; Colautti and Barrett, 2011; Bolstad et al., 2014; Houle et al., 2017; McGlothlin et al., 2018). An assumption of these analyses is that the observed **G** matrix is structurally similar to the ancestral **G** matrix that has shaped observed patterns of population and species divergence. Although recent studies suggest that the structure of the **G** matrix is often conserved during evolutionary divergence (Puentes et al., 2016; Houle et al., 2017; McGlothlin et al., 2022), the issue remains unresolved (Arnold et al., 2001; Arnold et al., 2008; Jones et al., 2014; Walter et al., 2018; Milocco and Salazar‐Ciudad, 2022). Thus, knowing the degree to which **G** is stable over evolutionary time is important, and additional studies linking individual **G** matrices to patterns of divergence can also contribute toward this goal.

Few previous studies have explicitly evaluated the evolvability of higher‐dimensional floral phenotypes. Opedal et al. (2017) developed methods for estimating the evolvability of composite traits, but limited their analysis to herkogamy in a single dimension. Flower morphology in many plant species deviates from linear orientation of male and female organs, including those species exhibiting movement herkogamy (where sexual organs move sequentially during anthesis; Ruan and da Silva, 2011), three‐dimensional heterostyly and reciprocity (*Linum*, Armbruster et al., 2006; *Oxalis*, Turketti et al., 2012; *Turnera*, Rech et al., 2020), and heteranthery (where anther shape and position vary within flowers; Vallejo‐Marín et al., 2009). Evaluating the evolvability of flower morphology in such species requires an alternative approach.

Here, we took a fully multivariate approach to quantifying the evolvability of three‐dimensional floral morphology. We analyzed the three‐dimensional structure of the sexual organs within *Dalechampia* blossoms in the light of known functional interactions among traits. First, we used data from a diallel crossing design in the greenhouse to estimate the genetic variance–covariance matrix for traits representing the relative positions of male and female sexual organs (anthers and stigmas). We then used the **G** matrix and the multivariate evolvability framework of Hansen and Houle (2008) to ask how genetic variation is distributed in multivariate space, and explicitly assess the influence of genetic constraints on evolutionary divergence by comparing patterns of population and species divergence to patterns of standing genetic variation in the same phenotypic directions.

## MATERIALS AND METHODS

### Study system

The tropical genus *Dalechampia* (Euphorbiaceae) has been a long‐term model system for investigating pollinator‐mediated floral evolution. The woody vine *D. scandens* L. s.l. is typical of the genus in having unisexual male and female flowers aggregated into functionally bisexual blossom inflorescences (Figure 1). In most *Dalechampia* species, including *D. scandens*, the pollinator reward is a sticky terpenoid resin secreted by a “gland” comprising aggregated bractlets associated with the male flowers. The three‐dimensional architecture of the blossom is of critical importance for pollination mechanics (Armbruster, 1988, 1990). The distance between the resin gland and the anthers of the male flowers (gland–anther distance, GAD) determines the minimum size of bee that will contact anthers while collecting resin, thus picking up pollen. Similarly, the distance between the resin gland and stigmas (gland–stigma distance, GSD) determines the minimum size of bees contacting stigmas and potentially depositing pollen. The two distances also influence where on larger pollinators pollen is deposited and stigmas make contact, respectively. The distance between anthers and stigmas (functional herkogamy, ASD) is strongly negatively correlated with autonomous self‐pollination and seed‐set in the absence of pollinators (Armbruster, 1988, 1993; Opedal et al., 2015, 2016a).

**Figure 2:**
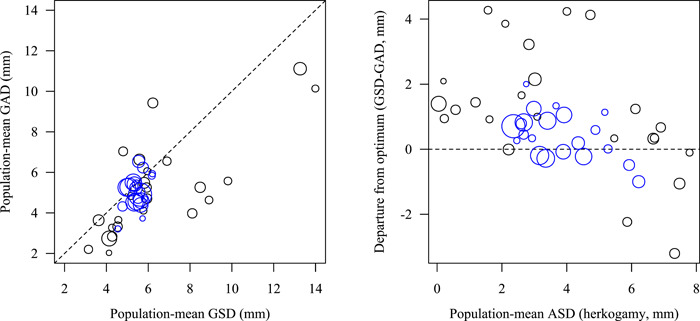
Graphical exploration of potential deviation from optimal morphology of *Dalechampia* blossoms. Across 45 populations of 12 species, population‐mean gland–stigma (GSD) and gland–anther distances (GAD) fall roughly along the isometric line, although there are exceptions. The deviation from the assumed optimum in terms of pollinator‐mediated pollen transfer (GSD = GAD) covaries with population‐mean anther–stigma distance (ASD, herkogamy), suggesting a conflict between optimality in terms of pollen transfer and herkogamy. Negative values for GSD – GAD indicate that anthers protrude beyond stigmas, presumably reducing autonomous self‐pollination. The size of the circles is proportional to the square root of the sample size. Blue circles indicate populations of *D. scandens* s.l. in Costa Rica.

These observations suggest that the adaptive optimum for pollinator‐mediated pollen transfer is where population‐mean gland–anther and gland–stigma distances are identical, thus ensuring pollen placement on that part of the bee's body that will contact the stigmas of subsequently visited blossoms (Armbruster et al., 2009a, 2009b). The optimum anther–stigma distance depends on the local optimum outcrossing rate and the importance of delayed self‐pollination (Opedal et al., 2016b; Hildesheim et al., 2019). Comparative studies have confirmed good correspondence between population‐ and species‐mean gland–anther and gland–stigma distances (Armbruster et al., 2009a), positive covariation between these traits and pollinator size (Armbruster, 1988), and positive covariation between anther–stigma distance and local pollination reliability (Opedal et al., 2016b). However, exceptions to these patterns (Figure 2) suggest that additional factors affect the joint evolution of these functionally important traits. The departure from optimum correspondence between gland–stigma distance and gland–anther distance (GSD = GAD) appears related to variation in anther–stigma distance, suggesting a potential conflict between optimum flower‐pollinator fit and optimum outcrossing rates. In more outcrossing taxa (as indicated by greater herkogamy) anthers tend to protrude beyond stigmas (GSD < GAD, e.g., *D. dioscoreifolia* in Figure 1) presumably reducing autonomous self‐pollination. In less outcrossing taxa, anthers tend to be located above the stigmas (GSD > GAD, e.g., *D. websteri* in Figure 1), promoting delayed self‐pollination.

### Diallel crossing design

To evaluate the evolvability of blossom morphology, we first used data from a diallel crossing design to estimate the genetic variance–covariance matrix (**G** matrix) for gland–stigma distance, gland–anther distance, and anther–stigma distance, three key traits that jointly describe the relative positions of male and female sexual organs (Figures 1 and 2). Seeds used in the greenhouse crossing design were collected in 2015 in a population close to the Estación Experimental Horizontes (10°41′54.4″N, 85°35′54.8″W) in Guanacaste, Costa Rica. The parental population was raised in the greenhouse in the spring of 2020. Each parental plant was grown from a seed from a separate infructescence, but seeds from several infructescences (assumed to be half‐sibs) were included for some of the maternal plants. Eight of the parental plants were derived from crosses among greenhouse‐grown plants grown from seeds collected in the field. We accounted for all known relationships among parental plants in the quantitative‐genetic analyses.

During April–July 2020, we crossed the paternal individuals following a block diallel design similar to that described by Hansen et al. (2003b). Each block consisted of four parental individuals originating from separate maternal individuals in the field. We performed all reciprocal crosses among the four members of each block, including selfing, for a total of 16 crosses per block and 160 crosses in total. This design yielded 40 half‐sib groups, some of which were also related to each other by sharing one grandparent.

During the late fall of 2020 and early spring of 2021, we raised up to three offspring from each cross, and measured blossom traits on two haphazardly chosen blossoms for each of two haphazardly chosen individuals. We measured gland–stigma distance as the average distance from the resin gland to the tip of each of the three stigmas, gland–anther distance as the distance from the resin gland to the anthers of the central male flower, and anther–stigma distance as the distance from the anthers of the central male flower to the closest stigma (cf. Figure 1). All measurements were taken on blossoms in the early bisexual condition (1–3 open male flowers). Due to a few failed crosses, germination failures, or flowering failure, the final sample size was 607 blossoms from 306 offspring from 39 half‐sib families.

### G‐matrix estimation

To estimate the **G** matrix, we fit a multivariate animal model with the MCMCglmm R package (Hadfield, 2010) to the form

$$z_{\mathrm{ijkl}}=\mu_{i}+a_{\mathrm{ij}}+b_{\mathrm{ij}}+d_{\mathrm{ik}}+q_{\mathrm{ijkl}},$$

where *z* is the phenotypic trait value, *μ* is the trait mean, *a* is the breeding value, *b* is the non‐genetic plant‐level effects, *d* is the measurement‐date effect, and *q* is the residual within‐plant effect. The indices run over traits (*i*), plants (*j*), dates (*k*), and individual blossoms (*l*). To estimate the **G** matrix (G), the “animal”‐level random effect was distributed as a~N(0,G⊗A), where A is the additive relatedness matrix derived from the breeding design, and ⊗ is the Kronecker product (which transforms the two matrices into a larger matrix containing the genetic covariances between individuals across all traits; Hadfield and Nakagawa, 2010). The additional random effects were included to account for non‐additive‐genetic sources of variation and estimate variance matrices among plants (b), measurement dates (d), and blossoms within plants (q). We sampled the posterior distribution for 750,000 iterations with a transient of 250,000 iterations discarded and a thinning interval of 500 and confirmed convergence by evaluating posterior trace plots and confirming effective sample sizes close to the expected 1000.

#### Measuring multivariate evolvability and constraint

Hansen and colleagues (Hansen et al., 2003a, 2019; Hansen and Houle, 2008) introduced a fully multivariate framework for quantifying evolvability of multivariate characters. The evolvability along a (hypothetical) selection gradient *β* is defined as the predicted evolutionary response to selection in the direction of *β*. To quantify multivariate constraints arising from the quantitative‐genetic architecture of complex phenotypes as represented by the genetic variance matrix G, Hansen et al. (2003a) also introduced the concept of conditional evolvability, defined as the predicted evolutionary response along *β* when other defined phenotypic traits are held constant (as when these are under stabilizing selection). In the present study, quantifying conditional evolvabilities allowed us to explicitly evaluate, for example, how much the realized evolvability of anther–stigma distance is reduced assuming stabilizing selection on the flower‐pollinator fit traits (gland–anther distance, gland–stigma distance). The proportion of genetic variance available while conditioning on the constraining traits is referred to as autonomy.

We used functions from the evolvability R package (Bolstad et al., 2014) to compute evolvabilities, autonomies, and conditional evolvabilities. We computed the mean (expected value) of each parameter across the **G** matrix and also the value of each along 1000 selection gradients randomly drawn from the unit sphere. These random selection gradients represent hypothetical ways in which selection could act on the multivariate phenotype, and this approach allowed us to assess the distribution of evolvabilities in multivariate phenotype space. Note that the mean evolvability along a large set of random uniformly distributed selection gradients approximates the mean evolvability across the **G** matrix, which is also equal to the expectation (mean) of the eigenvalues, and the mean of the univariate evolvabilities (the diagonal of the **G** matrix).

#### Phenotypic divergence of populations and species

To assess the role of genetic constraint in the evolution of floral architecture in *Dalechampia*, we assessed the relationships between evolvability (as quantified by the **G** matrix), population divergence, and species divergence following the approach of Bolstad et al. (2014). At the population level, we estimated the among‐population variance matrix $D_{P}$ for 16 Costa Rican populations measured in a common greenhouse environment (data from Opedal et al., 2016b). We fit a multivariate mixed model to the form

$$z_{\mathrm{ijkl}}=\mu_{i}+p_{\mathrm{ij}}+b_{\mathrm{ijk}}+q_{\mathrm{ijkl}},$$

where *z* is the phenotypic trait value, *μ* is the trait mean, *p* is the population effect, *b* is the within‐population plant‐level effect, and *q* is the residual within‐plant effect. The indices represent traits (*i*), populations (*j*), plants (*k*) and individual blossoms (*l*). The population‐level random effect estimates the variance matrix $D_{P}$ for population‐mean phenotypes across populations. The phenotypic measurements were ln‐transformed before analysis, and the estimated variances and covariances are therefore on a proportional scale comparable to the within‐population evolvabilities.

To estimate the among‐species variance matrix $D_{S}$, we fit a similar model to field measurements from 43 populations of 11 *Dalechampia* species (Appendix S1). Given the much greater divergence of species means compared to population means, we assumed that variation due to environment (plasticity) was negligible. This assumption is justified by the limited environmental variance in the study traits (Armbruster and Schwaegerle, 1996; Opedal et al., 2016a, 2016b). Most blossoms of one taxon belonging to the *D. scandens* complex (top left in Figure 1) have zero anther–stigma distance, which precludes ln‐transformation. Although this variation is biologically real, including this taxon had much leverage on the analysis by inflating the among‐species variance in anther–stigma distance. Therefore, we chose to exclude this taxon from the analysis, but note that including it would only make the observed patterns clearer.

The estimated **G** matrix describes proportional standing genetic variance within populations (“evolvability”), and the **D** matrices describe proportional variance among populations or species. The genetic‐constraints hypothesis predicts a positive relationship between proportional evolutionary divergence and evolvability (Bolstad et al., 2014; Houle et al., 2017). The expected slope of the relationship differs among distinct macroevolutionary models, but estimating the slope is complicated by estimation error in the **G** and **D** matrices (Houle et al., 2020 [Preprint]; Jiang and Zhang, 2020). Therefore, we assessed support for potential constraints by comparing G to $D_{P}$ and $D_{S}$ graphically (Bolstad et al., 2014). To do so, we computed and plotted evolvabilities, population divergence, and species divergence for the three original traits and along the 1000 randomly distributed unit‐length selection gradients. The latter approach allowed us to explore the evolvability–divergence relationship in higher dimensions, i.e., for combinations of the original traits. All analyses were performed in R 3.5.0 (R Core Team, 2018).

## RESULTS

Univariate evolvabilities differed markedly among the three original traits (Table 1), and the evolvability of anther–stigma distance (herkogamy) was much higher than the evolvability of the flower‐pollinator fit traits (gland–stigma distance, gland–anther distance). The very high average evolvability of the **G** matrix (i.e. the expected evolvability of a randomly chosen direction in phenotypic space) of 2.59% can be interpreted as the expected proportional response to an episode of unit‐strength selection and translates into an evolutionary doubling‐time of *t*_2_ = ln(2)/0.0259 = 26.8 generations under unit strength selection.

**Table 1:** The mean‐scaled **G** matrix for gland–anther distance (GAD), gland–stigma distance (GSD), and anther–stigma distance (ASD). Evolvabilities are given as a percentage (i.e., ×100) on the diagonal with 95% credible intervals in parentheses, additive genetic covariances above the diagonal, and genetic correlations below the diagonal.

Trait	GAD (95% CI)	GSD (95% CI)	ASD (95% CI)
GAD	0.409	0.193	0.309
	(0.247, 0.615)	(0.019, 0.377)	(–0.139, 0.761)
GSD	0.34	0.801	–0.591
		(0.486, 1.233)	(–1.300, 0.034)
ASD	0.19	–0.26	6.567
			(4.272, 9.437)

The moderately strong genetic correlations between the three focal traits translated into a median autonomy of 53.3% (Table 2), which means that about half of the unconditional genetic variance is available for independent evolution in a randomly chosen direction in phenotype space, i.e., a random combination of the original traits.

**Table 2:** Posterior means and 95% credible intervals for mean, minimum, and maximum evolvability ($\bar{e}$, $e_{\min}$, $e_{\max}$), mean conditional evolvability ($\bar{c}$), mean autonomy ($\bar{a}$), and mean integration ($\bar{i}$).

Parameter	Mean	Lower	Upper
Evolvability ($\bar{e}$)	2.592	1.766	3.551
Min evolvability ($e_{\min}$)	0.286	0.132	0.435
Max evolvability ($e_{\max}$)	6.640	4.325	9.549
Conditional evolvability ($\bar{c}$)	0.905	0.585	1.196
Autonomy ($\bar{a}$)	0.533	0.332	0.685
Integration ($\bar{i}$)	0.467	0.315	0.668

**Figure 3:**
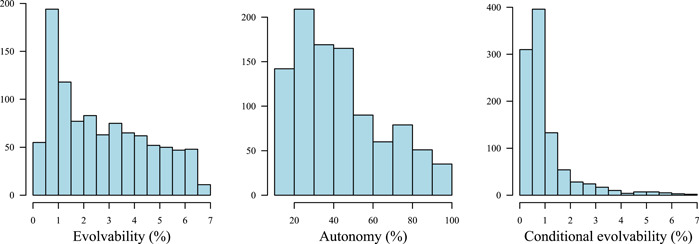
Distributions of multivariate evolvabilities, autonomies, and conditional evolvabilities along 1000 random unit‐length selection gradients (“traits”). Unconditional evolvabilities (“Evolvability”) describe the expected response in the direction of selection, autonomies describe the percentage of the unconditional evolvability that is independent of variation in other phenotypic directions, and conditional evolvabilities describe the expected response to selection while all orthogonal directions are kept constant (as when these are under stabilizing selection).

Exploring patterns of evolvability across randomly chosen directions in phenotypic space (Figure 3) revealed greater‐than‐average evolvability (>2.6%) for many directions in phenotypic space. In contrast, the skewed distribution of autonomies means that similarly high conditional evolvability was restricted to a smaller set of directions. Thus, for a considerable proportion of directions, evolutionary response would be much reduced in a scenario of stabilizing selection on orthogonal directions.

**Figure 4:**
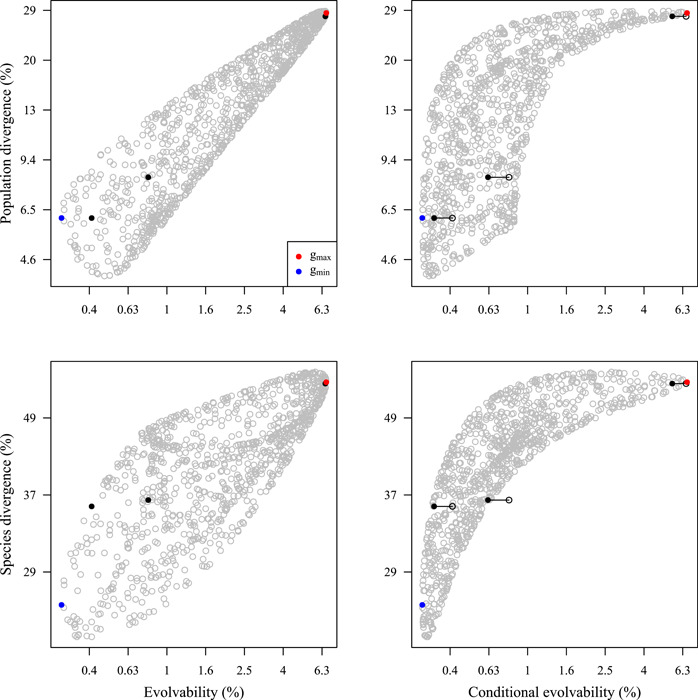
Relationship between evolutionary divergence (realized evolution) among populations and species and evolvability (evolutionary potential). Filled black symbols indicate the original traits (left to right) gland–anther distance, gland–stigma distance, anther–stigma distance; grey open symbols indicate 1000 random unit‐length selection gradients. The line segments on the conditional‐evolvability panels indicate the difference between the conditional (filled black symbol) and unconditional (open black symbol) evolvability for each trait. Note difference in *y*‐axis scales between upper and lower panels. The blue and red symbols indicate the least (*g*_min_) and most (*g*_max_) evolvable traits, as represented by the trailing and leading eigenvectors of the **G** matrix.

Comparing the extent of population divergence among the original traits and among directions in phenotypic space revealed an overall positive relationship, and the most evolvable focal trait (anther–stigma distance) has diverged the most among populations (Figure 4). Directions of high conditional evolvability exhibited more divergence than did directions of low conditional evolvability, yet low conditional evolvability did not necessarily limit divergence (Figure 4).

When focusing on the divergence of species and thus extending the time‐scale and extent of divergence, the positive relationships between divergence and evolvability remained. However, at the species level, both pollinator‐fit traits have diverged to approximately equal extents, despite greater evolvability of gland–stigma distance than gland–anther distance (Figure 4).

## DISCUSSION

Our analysis of multivariate evolvability reveals that *Dalechampia* blossom traits involved in flower‐pollinator fit and autonomous selfing exhibit appreciable independent evolutionary potential, but also that populations and species have diverged to a greater extent in those phenotypic directions associated with greater multivariate evolvability. The latter result is consistent with the hypothesis that joint trait evolution toward optimum flower–pollinator fit and optimum self‐pollination rate is subject to genetic constraints, thus possibly providing a partial explanation for the observed distribution of trait values across the genus (Figure 2; Armbruster et al., 2009a).

### Selection and genetic constraint in the evolution of flowers

The flowers of animal‐pollinated plants are complex structures, seemingly tuned to the tasks of attracting pollinators and transferring pollen to and from their bodies. When outcrossing fails due to pollinator shortage or other factors, self‐compatible species may compensate by self‐pollinating, which also requires specific floral mechanisms (Goodwillie and Weber, 2018; Hildesheim et al., 2019). When populations experience changes in pollinator communities either locally or during range expansion, flower phenotypes will often diverge in response to novel selection pressures associated with changes in flower–pollinator fit and the greater, or lesser, need for efficient self‐pollination (Moeller, 2006). Because flowers are at least partly integrated phenotypes (e.g., Anderson and Busch, 2006; Ordano et al., 2008; Armbruster and Wege, 2019), patterns of divergence may be constrained by genetic covariance among traits under conflicting selection pressures (e.g., herkogamy vs. traits affecting accurate pollen transfer). Consequently, populations may fail to track variation in several aspects of their environments simultaneously.

Comparative studies of pollinator relationships and mating systems in *Dalechampia* strongly suggest that populations and species are often well adapted to their principal pollinators (Armbruster, 1988) and to the local reliability of pollination (Opedal et al., 2016b). However, not all *Dalechampia* populations exhibit precise “matching”of traits important for pollen deposition and pick‐up (Figure 2, and see Hansen et al., 2000; Armbruster et al., 2009a), suggesting that other factors play a role in their evolution. For example, Costa Rican populations of *D. scandens* exhibit striking variation in herkogamy (Opedal et al., 2016b). In the most herkogamous (outcrossing) populations, gland–anther distances substantially exceed gland–stigma distances, suggesting that selection for avoidance of self‐pollination in these populations has led to departure from the optimal positions of anthers and stigmas with respect to where fertile parts contact the pollinators (Figure 2, blue circles). One hypothesis for why such “maladaptation” occurs in *Dalechampia* and in many other species (Hereford, 2009; Brady et al., 2019) is that conflicting selection and genetic covariance among functionally interacting traits reduce the rate at which populations track variation in their phenotypic optima.

Genetic constraints on evolution mean that the response to selection on any trait or combination of traits may be both reduced and deflected by genetically correlated traits under conflicting selection (Lande, 1979; Blows and Hoffmann, 2005; Hansen and Houle, 2008; Walsh and Blows, 2009; Futuyma, 2010). It has long been recognized that the genetic architecture of complex phenotypes may channel phenotypic evolution along specific directions in multivariate trait space associated with greater evolvability (Schluter, 1996; Chenoweth et al., 2010; Bolstad et al., 2014; Houle et al., 2017). Our analysis of evolvability and divergence across Costa Rican *D. scandens* populations (Figure 4) demonstrates greater divergence in those phenotypic directions associated with greater evolvability, consistent with the genetic‐constraints hypothesis. Positive evolvability–divergence relationships are emerging as a general pattern, as supported by similar findings for a different set of traits in Mexican *D. scandens* populations (Bolstad et al., 2014), and in other systems.

The form of genetic constraints assessed here can be seen as “soft constraints” in that they are thought to affect the rate at which a population can approach its optimum, rather than strictly precluding the approach of the optimum (Hansen and Houle, 2008; Futuyma, 2010). Indeed, some *Dalechampia* populations and species have evolved substantial herkogamy while maintaining pollination accuracy, as indicated by correspondence between male and female organ positions. Armbruster et al. (2009a) pointed out that those populations and species often do so by having male and female flowers diverging from one another at a substantial angle (Figure 1). Studies in other systems also demonstrate that the conflict between accuracy in pollen transfer and avoidance of self‐pollination (the “herkogamy dilemma” sensu Armbruster et al., 2009a) may be resolved, often through complete dichogamy (temporal separation of sexual functions; Lloyd and Webb, 1986) or other mechanisms such as movement herkogamy, as seen in *Parnassia* (Celastraceae; Armbruster et al., 2014), *Ajuga* (Lamiaceae; Ye et al., 2019), *Prepusa* (Gentianaceae; Lemos et al., 2020), and *Lysimachia* (Primulaceae; Jiménez‐López et al., 2020).

Finding such cases does not, in itself, provide evidence against the importance of constraints, however, because strong selection and long‐maintained, weaker selection would allow populations to approach their optima. For example, artificial‐selection studies have confirmed response to strong selection in directions of limited genetic variation (Conner et al., 2011). As one way to assess how the influence of genetic constraints changes over time, we expanded our focus to the interspecific level, analyzing divergence among species separated by tens of millions of years of divergence. The overall evolvability–divergence relationship held also at the species level, although the two pollinator‐fit traits had diverged about equally despite greater evolvability of gland–stigma distance. These different patterns at the population vs. species level could reflect reduced influence of genetic constraints on longer time scales, although it must be noted that the species‐level analysis was based on field measurements and the role of phenotypic plasticity cannot be conclusively ruled out.

The conserved evolvability–divergence relationship is consistent with previous work in *Drosophila* flies (Houle et al., 2017) and *Anolis* lizards (McGlothlin et al., 2018). These results also provide some justification for an important assumption of our study, that the **G** matrix estimated for a single population is structurally similar to the ancestral **G** matrix that has shaped observed patterns of population and species divergence. The relative stability of the **G** matrix is a largely unresolved issue in evolutionary biology, but our results add to an increasing number of studies suggesting long‐term preservation of **G** matrix structure (Puentes et al., 2016; Houle et al., 2017; McGlothlin et al., 2022).

An outstanding question is how the evolvability–divergence relationships among populations and species can be reconciled with the relatively high evolvability measured in our focal population, even after accounting for genetic covariances. For example, the greater univariate evolvability of herkogamy than of the flower–pollinator‐fit traits (gland–anther distance, gland–stigma distance) is consistent with a previous literature survey (Opedal et al., 2017) and with results from a Mexican population of *D. scandens* (Hansen et al., 2003b). The moderate genetic correlations translated into a multivariate autonomy of 84.8% for anther–stigma distance conditioned on the two fit traits, which means that the conditional evolvability of herkogamy in a scenario of stabilizing selection on the fit traits is still very high (*c* = 0.848*e* = 5.57%). This result confirms the unusually high evolvability of herkogamy (Herlihy and Eckert, 2007; Bodbyl Roels and Kelly, 2011; Opedal et al., 2017) and is inconsistent with strong constraints on adaptation to changes in the reliability of pollination. In general, conditional evolvabilities did not exhibit a more consistent relationship with population or species divergence in our analyses (Figure 4), possibly because estimates of genetic covariances and thus conditional evolvabilities are typically associated with high uncertainty (Sztepanacz and Blows, 2017).

Another source of uncertainty in analyses of evolvability and divergence is the dynamics of the adaptive landscape over time (Arnold et al., 2001; Hansen, 2012). For traits functionally involved in pollination, fluctuations in the adaptive landscape will often arise from temporal variation in pollinator assemblages (Albertsen et al., 2021; Opedal, 2021). The observed patterns of apparent maladaptation (Figure 2) combined with strong evolvability–divergence relationships (Figure 4) could represent a situation where environmental fluctuations happen too fast for populations to track. Specifically, given the greater evolvability of herkogamy compared to flower–pollinator fit traits, we propose that adaptation to fit morphology and behavior of local pollinators tends to lag behind adaptation to the reliability of local pollination. Such a scenario is consistent with current attention to fluctuating adaptive landscapes (Kopp and Matuszewski, 2014; de Villemereuil et al., 2020), especially if the measured (conditional) evolvabilities are upwardly biased, which is likely to be often the case (Bolstad et al., 2014).

Finally, the observed positive relationship between standing genetic variation and evolutionary divergence is consistent not only with the constraint hypothesis, but also with the alternative hypothesis that selection shapes variation within and among populations in similar ways (evolution along “selective lines of least resistance”; Arnold et al., 2001). These hypotheses are hard to separate empirically, although the selection model predicts alignment between evolutionary divergence and the inverse of the matrix of nonlinear selection within populations (**γ**). One way forward would thus be to combine estimates of population divergence, (multiple) **G** matrices, and multiple selection estimates (Chenoweth et al., 2010). Unfortunately, these types of data are rarely available for the same empirical system, especially for plants.

### Multivariate evolvability of flowers

Most studies assessing genetic constraints on floral evolution have focused on heritabilities and bivariate genetic correlations (e.g., Ashman and Majetic, 2006 and references therein). Even when the multivariate evolvability of flowers has been considered, it has only rarely been assessed through multivariate analysis of **G** matrices (but see Smith and Rausher, 2008; Bissell and Diggle, 2010). Jiménez‐López et al. (2019) considered higher‐dimensional genetic variation in *Lysimachia arvensis*, which is unusual in combining “lateral” herkogamy through an angle between pistil and stamens during early flower ontogeny with “vertical” herkogamy following movement of the stamens into the axis of the pistil. Although this analysis was not strictly multivariate, it did suggest considerable evolvability of higher‐dimensional floral morphology.

Just as our understanding of the evolutionary potential of univariate traits has changed when mean‐scaled instead of variance‐scaled genetic variances have been employed (Houle, 1992; Hansen et al., 2011; Opedal, 2019), we suspect that there is much to be learned by moving from bivariate correlations to multivariate autonomies (and conditional evolvabilities) as measures of constraint (see also Conner, 2012). Our approach to studying multidimensional evolvability extends readily to even higher dimensions than considered here. For example, the increasing availability of 3D‐morphometric data for flowers (van der Niet et al., 2010; Dellinger et al., 2019; Reich et al., 2020), should provide scope for assessing evolvability across tens of dimensions. A challenge in such studies will be to estimate large **G** matrices, although methods such as genetic principal component analysis (Kirkpatrick and Meyer, 2004) and Bayesian latent factor analysis (Runcie and Mukherjee, 2013) should greatly facilitate this work. Work on fruit flies suggest that, for high‐dimensional phenotypes, a reduced subset of dimensions (as represented by, e.g., principal components) harbor detectable genetic variation, with the remaining dimensions constituting a “nearly null subspace” harboring almost no variance (Gomulkiewicz and Houle, 2009). Flowers are often considered to be highly modular, but it remains unknown how genetic variation is distributed in trait space.

We propose that further progress in the understanding of floral evolvability is likely to arise from adopting a multivariate approach explicitly considering trait covariances. As we have stressed throughout, however, the choice of focal traits to include in the **G** matrix needs to represent knowledge about the functional relationships between traits, performance, and fitness (Conner, 2007; Opedal, 2021). Indeed, the insights of our analyses could not have been reached without merging theoretically relevant analytical methods with in‐depth knowledge of trait function and natural history.

## AUTHOR CONTRIBUTIONS

Ø.H.O. conducted field and greenhouse work, curated and analyzed data, crafted figures, and wrote the first draft of the manuscript. W.S.A. conducted fieldwork, L.S.H. conducted greenhouse work, and all authors contributed to manuscript revisions.

## Supporting information


**Appendix S1**. Trait means (± standard error) of *Dalechampia* taxa included in the analysis of species divergence.

## Data Availability

Data and R code are available at GitHub: github.com/oysteiop/Evolvability3D (https://doi.org/10.5281/zenodo.3712825).
