# Supplementary material for: Evolvability and constraint in the evolution of three‐dimensional flower morphology
Source: Am J Bot. 2022 Nov 13;109(11):1906–17. doi: 10.1002/ajb2.16092 (PMC9827957; doi:10.1002/ajb2.16092)
Supplement: Supplementary file 1 — Appendix S1. Trait means (± standard error) of Dalechampia taxa included in the analysis of species divergence. [file AJB2-109-1906-s001.pdf]

Evolvability and constraint in the evolution of three-dimensional flower morphology

Øystein H. Opedal[1,*], Laura S. Hildesheim[1] & W. Scott Armbruster[2,3]

[1]Department of Biology, Lund University, Sölvegatan 37, 22362 Lund, Sweden

[2]School of Biological Sciences, University of Portsmouth, Portsmouth PO1 2DY, UK

[3]Insitute of Arctic Biology, University of Alaska Fairbanks, Fairbanks, AK 99775, USA

*Corresponding author: oystein.opedal@biol.lu.se

SUPPORTING INFORMATION

Appendix S1

Table S1. Trait means (±standard error) of *Dalechampia* taxa included in the analysis of species divergence.

Table S1. Trait means (±standard error) of *Dalechampia* taxa included in the analysis of species divergence. GSD = gland-stigma distance, GAD = gland-anther distance, ASD = anther-stigma distance. Sample sizes are given for number of populations (n_{pop}), and number of blossoms (n).

Species	n_{pop}	n	GSD (mm)	GAD (mm)	ASD (mm)
D. aristolochiifolia H.B.K.	8	63	5.81 ±0.12	5.94 ±0.18	6.80 ±0.16
D. dioscoreifolia Poepp.	3	13	6.27 ±0.29	9.54 ±0.23	8.16 ±0.68
D. heteromorpha Pax & K.Hoff.	3	7	4.54 ±0.16	3.93 ±0.31	1.67 ±0.22
D. osana Armbr.	3	13	7.27 ±0.71	4.73 ±0.20	3.11 ±0.72
D. scandens A	23	380	5.41 ±0.05	5.05 ±0.05	3.56 ±0.08
D. scandens B	7	54	4.07 ±0.11	2.74 ±0.08	0.36 ±0.09
D. scandens C	1	12	3.63 ±0.17	3.64 ±0.19	2.21 ±0.28
D. scandens D	2	6	4.41 ±0.44	3.37 ±0.38	2.82 ±0.60
D. sp. nov. *'bella'*	1	5	5.77 ±0.58	4.11 ±0.45	2.61 ±0.45
D. sp. nov. *'leucocarpa'*	1	9	8.11 ±0.37	3.98 ±0.19	4.73 ±0.50
D. tiliifolia Lam.	2	21	13.44 ±0.39	10.88 ±0.23	2.80 ±0.34
D. websteri Armbr.	2	17	8.95 ±0.29	5.37 ±0.28	3.25 ±0.34

The taxa denoted as *D. scandens* A-D are distinct but yet undescribed taxa.
